# Evaluation of Vetiver Volatile Compound Production under Aeroponic-Grown Conditions for the Perfume Industry

**DOI:** 10.3390/molecules27061942

**Published:** 2022-03-17

**Authors:** Carole Gavira, Françoise Watteau, Jean-Marc Lainé, Frédéric Bourgaud, Laurent Legendre

**Affiliations:** 1 Plant Advanced Technologies (PAT), F-54500 Vandoeuvre-lès-Nancy, France; jeanmarc_laine@bbox.fr (J.-M.L.); frederic.bourgaud@plantadvanced.com (F.B.); 2 Laboratoire Sols et Environnement (LSE), UAR 3562 CNRS INRAE, Université de Lorraine, F-54000 Nancy, France; francoise.watteau@univ-lorraine.fr; 3 Laboratoire d’Ecologie Microbienne, UMR CNRS 5557 INRAE 1418, Université Claude Lyon 1, F-69622 Villeurbanne, France; laurent.legendre@univ-lyon1.fr

**Keywords:** *Chrysopogon zizanioides*, volatile organic compounds, root microstructure, root ontogeny, HS-SPME GC/MS, soilless culture

## Abstract

Vetiver (*Chrysopogon zizanioides* (L.) Roberty) is a major tropical perfume crop. Access to its essential oil (EO)-filled roots is nevertheless cumbersome and land-damaging. This study, therefore, evaluated the potential of vetiver cultivation under soilless high-pressure aeroponics (HPA) for volatile organic compound (VOC) production. The VOC accumulation in the roots was investigated by transmission electron microscopy, and the composition of these VOCs was analyzed by gas chromatography coupled with mass spectrometry (GC/MS) after sampling by headspace solid-phase microextraction (HS-SPME). The HPA-grown plants were compared to plants that had been grown in potting soil and under axenic conditions. The HPA-grown plants were stunted, demonstrating less root biomass than the plants that had been grown in potting soil. The roots were slender, thinner, more tapered, and lacked the typical vetiver fragrance. HPA cultivation massively impaired the accumulation of the less-volatile hydrocarbon and oxygenated sesquiterpenes that normally form most of the VOCs. The axenic, tissue-cultured plants followed a similar and more exacerbated trend. Ultrastructural analyses revealed that the HPA conditions altered root ontogeny, whereby the roots contained fewer EO-accumulating cells and hosted fewer and more immature intracellular EO droplets. These preliminary results allowed to conclude that HPA-cultivated vetiver suffers from altered development and root ontology disorders that prevent EO accumulation.

## 1. Introduction

With an annual industrial production that has been estimated at approximately 250 t per annum, vetiver (*Chrysopogon zizanioides* (L.) Roberty) is a major tropical perfume crop [1]. The plant is a tall, perennial Indian bunchgrass belonging to the Poaceae family that develops dense tufts. Its roots contain an essential oil (EO) collected by steam distillation and finds wide applications in the perfume and cosmetic industries due to its fixative, fragrance, and skincare properties [2]. Pharmaceutical, agrochemical, and food and beverage industry applications have also been described due to the multifunctional bioactivities of vetiver EO that were recently reviewed [3,4].

Vetiver EO has a unique fragrance that is characterized by a long-lasting woody note and a fresh, herbal top-note [5]. Its composition is complex and has been scrutinized for many years. It is composed of more than 300 substances, most of which are sesquiterpenes that may be hydrocarbons or carry alcohol, aldehyde, ketone, or acid moieties [3,6]. Some terpenic ketones (ziza-6(13)-en-3-one, 2-epi-ziza-6(13)-en-3-one, β-vetivone, α-vetivone and khusimone) and alcohols (khusimol, (*E*)-isovalencenol and zizaen-3α-ol) collectively contribute to the global fragrance of vetiver [7]. Among them, 2-epi-ziza-6(13)-en-3-one has recently been shown to be the key compound responsible for the woody-ambery note specific to vetiver EO [8]. In contrast, terpenic acids (such as zizanoic acid) impair EO quality and make it unsuitable for perfumery applications [9], similar to (–)-geosmin, which is potentially associated with the undesirable earthy tonality of vetiver EO [7,8]. Geographic origin and extraction methodology have been shown to influence EO yield and composition [10,11].

Soilless crop production in controlled greenhouse environments has recently received much attention, as it may be able to help sustain a growing world demand for food in the context of water and arable land scarcity as well as climate havoc and a need to lower the environmental impact of agriculture [12,13,14]. Among the multitude of soilless production systems, high-pressure aeroponics (HPA) are relatively recent and have received the greatest amount of interest in the literature [15]. With this technology, the roots are kept bare in a humid atmosphere, and they are regularly sprayed with a very fine mist that is generated from a pressurized nutrient solution. This method is of particular interest when easy access to the roots is needed or when the artificial substrates used for soilless culture are banned to prevent them from impacting the environment through their disposal or production [15,16]. Noteworthily, HPA-based production of plant-specialized metabolites has found successful applications in the cosmetic and pharmaceutic industries with, for example, the production of prenylated polyphenols [17] and plant-based vaccines [18]. Several studies on the soilless cultivation of medicinal and aromatic crops have demonstrated advantages in terms of biomass yield and the quality of active chemical compounds [19,20,21]. Only one study has reported on the quality and quantity of EO from vetiver grown in soilless conditions [22]. A Thai vetiver (ecotype Mae Hae) was cultivated under semi-hydroponic conditions and was found to have a high EO yield with highly volatile constituents, similar to those of an EO produced from plants grown in parallel in traditional potting soil. The overall yield was only a third lower, and the rest of the constituents (the less volatile ones) were not analyzed. This study is, nevertheless, encouraging because the soilless vetiver cultivation does not require the tedious uprooting of massive vetiver plants or the associated soil erosion and loss of live plant-associated agronomic benefices, such as the protection of neighboring crops from pests and the long-term production of leaves for feed [23]. Nevertheless, semi-hydroponic cultivation is still unable to provide quick access to a clean root system that is ready for distillation.

Therefore, the aim of this study was to evaluate the potential of HPA-based vetiver cultivation to produce volatile organic compounds (VOCs) for the perfume industry. Plants were, therefore, cultivated in parallel under HPA and in a traditional potting soil growing medium. The root biomass and root system architecture were assessed for both plant groups, as well as the root VOCs. Root VOCs were sampled by headspace solid-phase microextraction (HS-SPME) and analyzed by gas chromatography coupled with mass spectrometry (GC/MS). To provide a rationale for the observed inability of the HPA-grown plants to accumulate most of the major VOCs constituents, the ultrastructural characteristics of the roots were unveiled for both plant groups via transmission electron microscopy (TEM) and light microscopy of dye-stained root sections. Root development and VOC accumulation of axenic, in vitro-grown plants, were also assessed to determine the potential role played by root-associated microbes.

## 2. Results and Discussion

### 2.1. Impact of HPA Cultivation on Root Biomass and VOCs Accumulation

**Figure 1:**
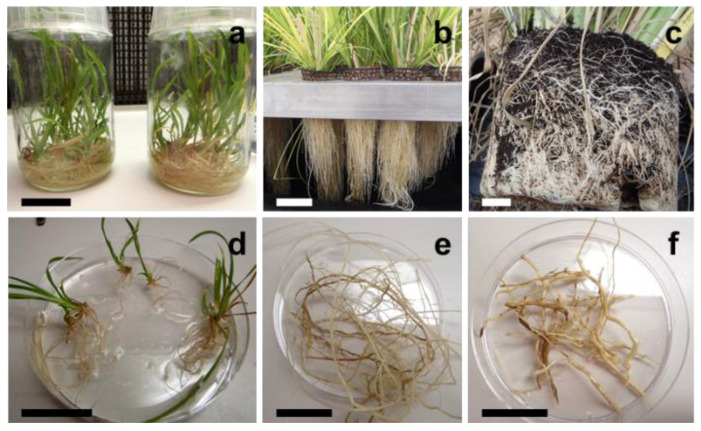
(**a**–**c**) Root systems and (**d**–**f**) root samples of vetiver grown (**a**,**d**) in vitro, (**b**,**e**) in high-pressure aeroponics (HPA), or (**c**,**f**) in potting soil. Bars represent (**a**) 3 cm, (**b**) 10.5 cm, (**c**) 4.8 cm, and (**d**–**f**) 3 cm.

The roots of HPA-grown plants were markedly smaller than those grown in potting soil (Figure 1). After four months of culture, they reached their maximum and had achieved stable development. The air-dried root biomass of the maximum-sized single plants was approximately 25 times smaller for the HPA-grown plants (an average of 1.2 ± 0.6 g as opposed to 30.4 ± 13.6 g). The root system was less developed and was made of longer, thinner, and more tapered roots. Even after dehydration with alcohol or acetone, the soil-grown (SG) plants displayed larger root diameters (up to 1500 µm as opposed to 250–500 µm).

The biomass could be stimulated via the addition of growth regulators. The use of brassinosteroid analogs on a hydroponic vetiver culture was reported [24], but it did not affect the dry matter of the vetiver roots.

**Figure 2:**
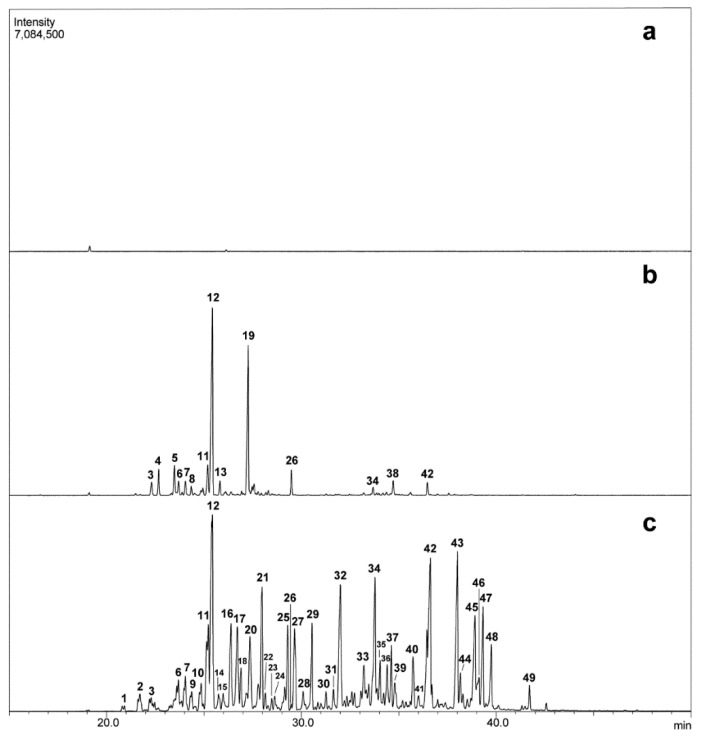
Headspace solid-phase microextraction and gas chromatography coupled with mass spectrometry (HS-SPME GC/MS) analysis of vetiver root volatile organic compounds (VOCs). Plants were grown (**a**) in vitro, (**b**) in HPA or (**c**) in potting soil.

A GC/MS-based analysis of the HS-SPME-sampled root VOCs of SG plants revealed a complex profile of 43 peaks (Figure 2). The substances making up most of the major peaks could be identified based on a comparison of their mass spectrum and retention index with reference substances that were previously entered in databases (Table 1). Most of them were hydrocarbon and oxygenated sesquiterpenes, with the most abundant substances being khusimene (12), δ-amorphene (21), epi-zizanone (34), khusimol (42) and (*E*)-isovalencenol (43). An identical VOC profile was obtained during the parallel analysis of an independent SG plant that originated from a different pot. These VOC profiles were also similar to those observed in a previously reported EO composition developed for commercial vetiver plants [10], despite the different methodologies used for VOC extraction (HS-SPME vs. steam distillation).

In contrast, the same VOC analysis that was conducted on the HPA-grown vetiver roots revealed a much simpler chemical blend (Figure 2 and Table 1). Even though it was also dominated by khusimene (12) and shared most of the relatively unabundant and more volatile hydrocarbon terpenes, it was devoid of most of the less volatile hydrocarbon and oxygenated sesquiterpenes that composed most of the total TIC trace area of the MS chromatogram of the VOCs that were sampled via HS-SPME from the SG plants. More surprisingly, the only other abundant VOC found in the HPA-grown vetiver roots was isodaucene (19), a substance that was not seen in our SG plant root extracts and that has not yet been reported in vetiver. Similar analyses carried out on independent four-month-old HPA-grown plants in May and November 2015 generated analogous GC/MS profiles. This daucane-type sesquiterpene is present in a dozen plant families (Apiaceae [25,26], Araceae [27], Asteraceae [28,29,30], Cordiaceae [31], Cupressaceae [32], Myrtaceae [33], Rosaceae [34], Rutaceae [35] and Zingiberaceae [36]), but this is the first mention of it in Poaceae. Unsurprisingly, the distortion in the VOC profile of HPA-grown plants was associated with an absence of the typical vetiver fragrance. The quantity of the hydrodistilled EO was also very low: 0.017% DW as opposed to 0.70% DW, 0.63% DW and 0.30% DW for field plants grown in Kenya, Senegal and France (Reunion Island), respectively. The qualitative and quantitative modifications that take place during EO production and that are induced by aeroponic culture conditions have already been observed. An example is described by Sainz and her coauthors, who observed differences in *Artemisia pedemontana* depending on cultivation methods (aeroponically vs. greenhouse) [37].

At first glance, the results of this study appear to differ from the conclusions reached by a previous analysis [22] of vetiver plants grown under semi-hydroponic conditions, in which the EO content was only reported to be one-third of the EO content measured in SG plant roots. Nevertheless, only the most volatile root substances were analyzed: a group of substances for which little difference was seen in the present semi-quantitative study (similar GC/MS trace profiles between SG and HPA-grown plant roots with khusimene (12) as the most abundant constituent).

In conclusion, the vetiver cultivation under HPA massively impaired the accumulation of the less volatile hydrocarbons and oxygenated sesquiterpenes in its roots, although they made up most of the HS-SPME-sampled VOCs found in SG plants. This resulted in a large diminution in the total content of the accumulated root volatiles and EO. This loss of EO biosynthesis was exacerbated by the lower root biomass of the HPA-grown plants.

### 2.2. Impact of a Root-Associated Microbiome on Vetiver VOC Accumulation

**Figure A1:**
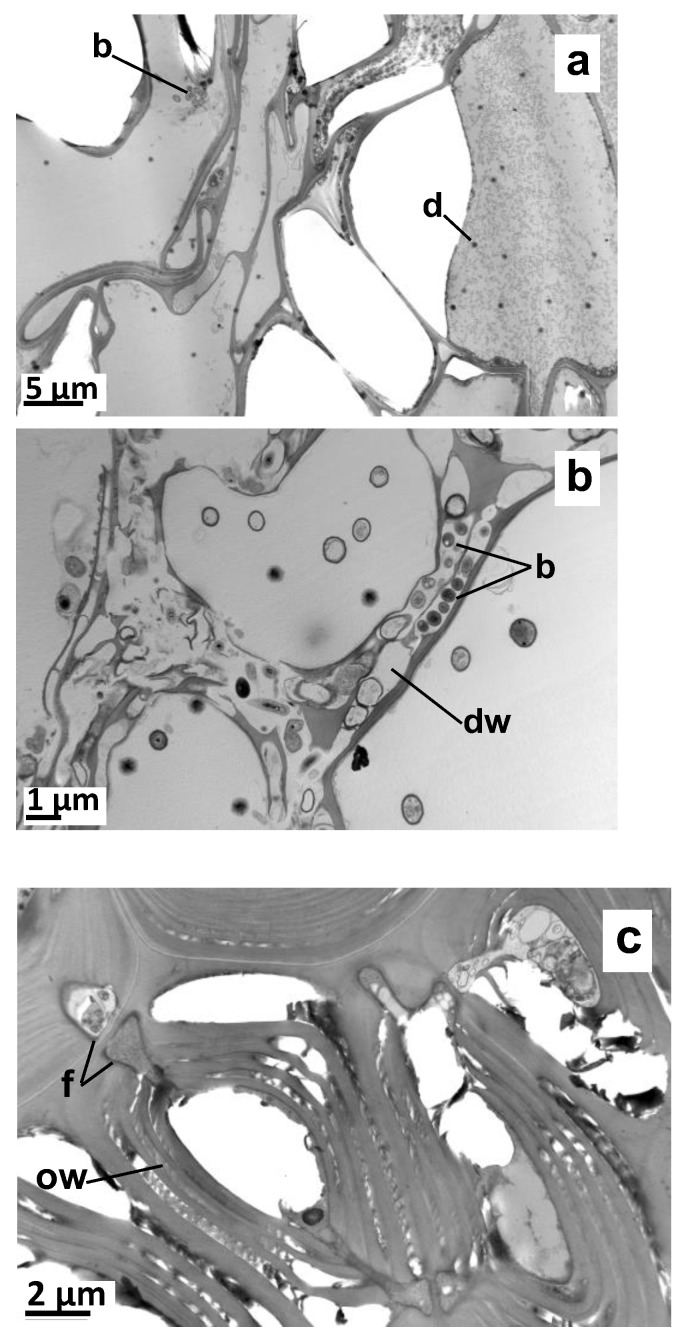
TEM views of microorganisms in root sections. (**a**) Bacteria near the cortical parenchyma cells in aeroponic plant roots. (**b**) Bacterial lysis in root cell walls of potted plants. (**c**) Fungi in ligneous parenchyma cells of potted plants. b: Bacteria; d: EO droplet; f: fungus; dw: degraded cell wall; ow: onion-like structure of the cell wall.

Root-associated microbes have been suggested to participate in the biosynthesis of the rich terpene diversity that accumulates in vetiver roots by metabolizing certain vetiver-synthesized hydrocarbon sesquiterpenes into more diverse structures [41]. The HPA-grown and the SG plants used in this study both originated from plants that had been grown in soil in the same greenhouse. They, therefore, originally shared a similar root-associated microbiota before being subjected to different selection pressures created by their different growing conditions. A TEM analyses of the ultrathin root sections confirmed that the roots of both the HPA-grown and SG plants hosted bacteria (Appendix A Figure A1). Nevertheless, the fungi and lytic zones within the ligneous cell walls were only visible in the SG plants.

To assess whether the absence of the association of key microorganisms with HPA-grown plant roots was responsible for the absence of the accumulation of some volatile compounds, vetiver plants were tissue-cultured under sterile conditions. These in vitro plants exhibited long and slender roots that resembled those of the HPA-grown plants (see Figure 1). Once dehydrated with alcohol or acetone, their diameter was approximately 250 µm, i.e., similar to those observed in the HPA-grown plants with the thinnest roots. The HS-SPME samples of the root VOCs were analyzed using GC/MS under the same conditions as those implemented for the HPA-grown and SG plants. Nevertheless, no substance was detected (see Figure 2). Since microorganisms are only responsible for the modification of vetiver sesquiterpenes at best and are most likely not responsible for the direct biosynthesis of all vetiver terpenes [41], the lack of accumulation of most sesquiterpenes in the HPA-grown plants is probably not due to a deficient microbiome. This may, however, result from the root ontogeny differences that are associated with culture conditions.

### 2.3. Impact of Culture Conditions on Root Development

**Figure 3:**
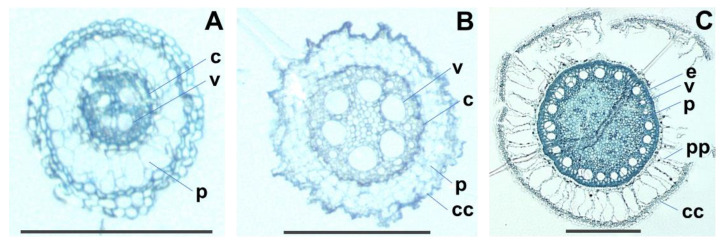
Toluidine blue-stained cross-sections of vetiver root. Plants were grown (**A**) in vitro, (**B**) in HPA, or (**C**) in potting soil. Observations were made using a photonic microscope. Bars represent 250 μm. c: central cylinder; cc: cortical cells; e: endoderm; p: parenchyma; pp: palissadic parenchyma; v: xylem vessel.

A large body of work has established many plant species whose root architectures are dramatically impacted by soil compaction [42,43]. This notably leads to shorter and thicker roots, a phenomenon that has been well described in many monocotyledonous species, such as in wheat [44] and other cereals [45]. According to our results, vetiver reacts similarly since the HPA-grown and in vitro-grown plants that were grown without substratum or that were grown on a soft substrate, respectively, had longer and thinner roots than the SG plants did (see Figure 1). Toluidine blue-stained thin cross-sections revealed that the SG plants had more robust roots since these were less prone to collapse during section preparation than those from the HPA-grown and in vitro-grown plants. They had a thicker cortex and a larger central cylinder that contained more vascular tissue and a larger internal pith (Figure 3). The in vitro-grown plant roots had a slightly less abundant cortex, vascular tissue and pith than those observed when grown under HPA, a finding that is in agreement with their smaller diameter.

Unfortunately, the toluidine blue-staining of root cross-sections did not allow for the visualization of EO droplets under the photonic microscope. Even Sudan red staining did not yield clear results despite the ability of this dye to specifically stain EO droplets in plants [46]. It is possible that the presence of the fixing resin impaired dye diffusion through the root sections. TEM was, therefore, conducted to locate the EO droplets in the ultrathin sections.

**Figure 4:**
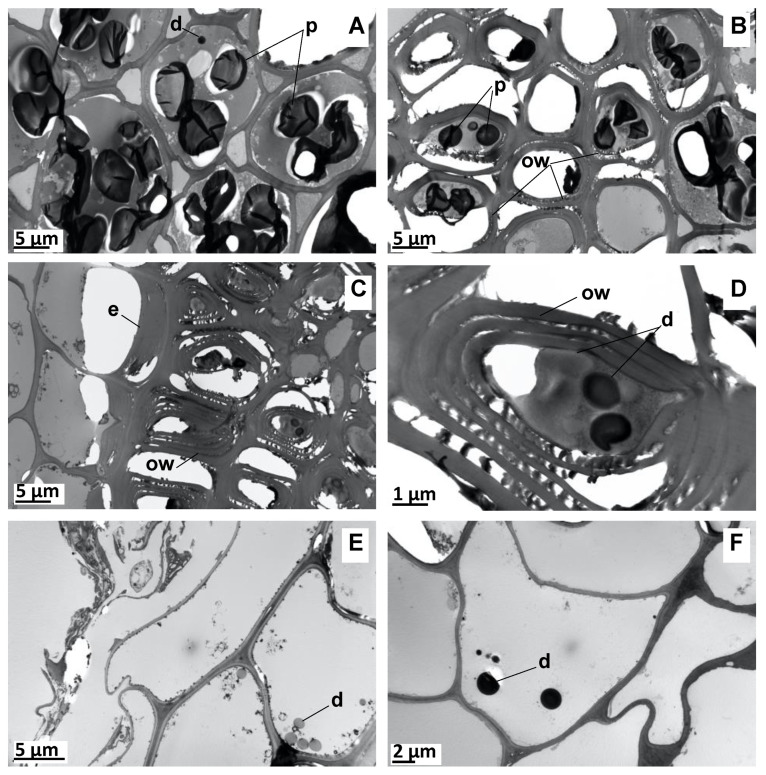
Transmission electron microscopy (TEM) of vetiver root grown in potting soil. (**A**,**B**) Woody parenchyma cells of the central cylinder. (**C**,**D**) Endoderm and adjacent tissue. (**E**,**F**) Cortical parenchyma cells. d: EO droplet; e: endoderm; ow: onion peel-like structure of the cell wall; p: essential oil (EO) pocket.

The TEM of the mature SG vetiver roots revealed the presence of intracellular EO droplets that were 1–2 µm in diameter and characterized by a smooth and electron-contrasted appearance. These were present in three different locations: in the wood parenchyma (pith), next to the endodermis, and within the cortical parenchyma (Figure 4). Several EO pockets often co-occurred within single cells. These could be larger and were able to reach 8 µm in diameter within the woody parenchyma cells. These results agree with previously published localizations of vetiver EO in intracellular vesicles in the root phloem region and in the pith [47,48], in the cortical parenchyma located just above the endodermis and, to a lesser extent, in the lysigen lacunae of the cortex [2,49,50].

**Figure 5:**
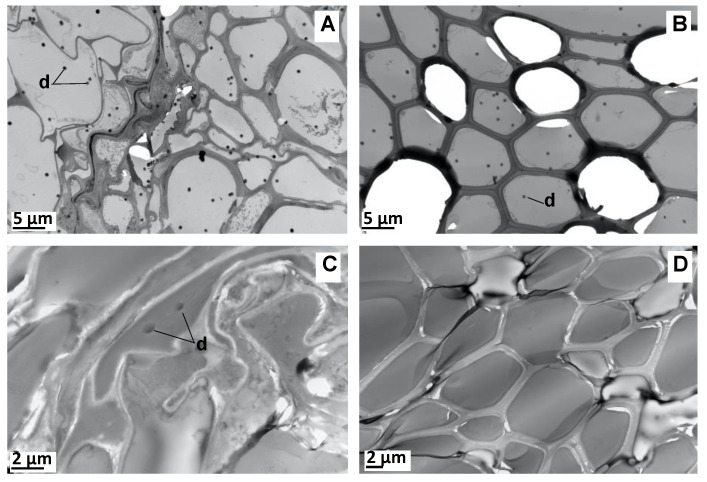
TEM of HPA-grown vetiver root cross-sections. (**A**) Stained section of parenchyma cells. (**B**) Stained section of woody cells. (**C**) Unstained section of parenchyma cells. (**D**) Unstained section of woody cells. d: EO droplet.

No similar EO vesicles were observed during the TEM analysis of the HPA-grown plant roots (Figure 5). Instead, less than one micrometer-wide droplets with strong electron contrast were seen across the cortical and woody parenchyma. The parallel observations of the unstained sections only revealed the presence of such vesicles within the cortical parenchyma cells (Figure 5C,D). This suggests that section staining led to vesicle spreading across the entire root section from their original location in the cortical parenchyma cells. The same observations were undertaken in the in vitro-grown plants, although they only contained rare EO vesicles (data not shown).

Overall, these results suggest that the vetiver cultivation under HPA-growth conditions leads to an altered root ontogeny, whereby the roots are slenderer and have fewer EO-accumulating cells that accumulate fewer intracellular droplets. These do not undergo full maturation since they do not swell in parallel with the weaker accumulation of detectable VOCs. The parallel analysis of the in vitro-grown plants confirmed this conclusion, as they exhibited more pronounced root ontogeny mishap trends, with even fewer EO droplets containing cells, fewer and thinner intracellular EO droplets, and a parallel absence of VOCs.

Some modifications to the soilless cultivation methodology proved to be able to rescue the retardation observed in the root ontology induced by standard soilless conditions in some other plant species. A hydroponic culture with an air gap above the nutrient solution was indeed shown to accelerate the maturation of the second exodermal layer of *Iris germanica* roots [51]. Similarly, an aeroponic growing unit with a separate compartment above the spray nutritive chamber allowed for the development of the *Zingiber officinale* rhizome [52]. Considering these results and those of this study, it would be interesting to assess whether such cultivation practices would resuscitate root development, intracellular EO vesicle maturation, and EO accumulation in vetiver.

## 3. Materials and Methods

### 3.1. Plant Material and Culture Conditions

Tufts of mature *C. zizanioides* were grown in pots filled with a regular horticultural growing medium in greenhouses at Plant Advanced Technologies (PAT, Vandoeuvre-lès-Nancy, France). Once the pots were overgrown, small clumps of 2 to 3 tillers were split off and were used to seed new pots with fresh potting medium. No pot-to-pot differences were seen in the VOC profiles of plants (data not shown). Small, detached clumps of 2 to 3 tillers of mature plants were also used to establish parallel HPA cultures, while individual tillers were used to initiate in vitro cultures.

To initiate HPA cultures, the roots and aerial parts of the freshly collected tillers were cut back to 20 cm. Plant-Prod^®^ from Fertile (Boulogne-Billancourt, France) containing nitrogen, phosphorus, and potassium ratios of 15, 10 and 30, respectively, was used as a nutritional medium. The electroconductivity of the medium was maintained between 1.0 and 1.2 mS cm^−1^, and the pH was maintained between 5.8 and 6.2. These 2 parameters were adjusted, if necessary, once a week. The HPA-grown plants were grown for at least 4 months in the same greenhouse as potted plants at PAT (Vandoeuvre-lès-Nancy, France) before assessment. By this time, they had reached their maximum and stable size under such growing conditions.

For the in vitro experiments, MS medium [53], purchased from Duchefa (Haarlem, The Netherlands) supplemented with 3% sucrose, 250 µg L^−1^ biotin, 500 µg L^−1^ folic acid, and 0.7% agar for solid media was used with its pH adjusted to 5.8. To initiate the in vitro cultures, pieces of stem, including the bud (5 cm long), were first sterilized in 70% ethanol for 1 min and then for 15 min in a 1.25% sodium hypochlorite solution containing 2 to 4 drops of Tween^®^ 20 per 40 mL of solution. After being rinsed generously 3 times in sterile water, the buds were grown aseptically on a solid MS medium enriched with 5 mg L^−1^ riboflavin, 500 µg L^−1^ zeatin, and 500 µg L^−1^ polyvinylpyrrolidone, and the macro-element concentration was divided by 2. The culture was incubated for 1 month at 23 °C during the daytime and at 21 °C overnight without light. The apexes of the newly formed stems were sampled and grown on the same medium devoid of polyvinylpyrrolidone for an additional month with a 16 h photoperiod. This led to the formation of shoots that were propagated for 1 month on a solid MS propagation medium containing 1 mg L^−1^ benzyl adenine and 6-(γ,γ-dimethylallylamino)purine. Whole plants were obtained by cultivating individual shoots for 1 month on liquid rooting MS medium enriched with 900 µg L^−1^ thiamin, and the macro-element and micro-element concentrations were divided by 4. The medium was refreshed monthly for at least 4 months.

### 3.2. Microscopy

Fifteen root segments (5 mm long) were sampled from the middle part of the roots of 2 full-size plants that were either grown in potting soil, aeroponically, or in vitro. They were immediately fixed with glutaraldehyde (2.5% *w*/*v* in cacodylate buffer pH 7.2) for 16 h and were further fixed with osmium tetroxide (2% *w*/*v* in cacodylate buffer pH 7.2) for 1 h. The samples were rinsed with cacodylate buffer after each fixing phase. Then, a vacuum was created to eliminate the air bubbles trapped inside the plant tissues. Doubly fixed samples were dehydrated in either graded alcohol or acetone to avoid the dissolution of the EO constituents. Finally, the dehydrated samples (5 per plant) were gradually embedded in epoxy resin (Epon 812) until complete polymerization was achieved (16 h at 60 °C). Thin (150 nm) and ultrathin (80–100 nm) sections were cut with a diamond knife on an Ultracut S ultramicrotome (Leica Microsystems, Nanterre, France). Thin sections were stained with toluidine blue and were examined with a photonic microscope (Leica Microsystems, Nanterre, France). Ultrathin sections were laid on nickel grids and were stained with uranyl acetate and lead citrate to enhance the organic matter contrast. They were then examined on a JEM 1200 EXII TEM (JEOL, Tokyo, Japan) operating at 80 kV.

### 3.3. VOC Analyses

The same lots of plants as those used for microscopy imaging were subjected to accumulated VOC analyses. To obtain samples with sufficient biomass, the roots of several in vitro-grown and HPA-grown plants were pooled into single samples. For each growing condition, at least 2 samples that had been generated from independent sets of plants or from different times were analyzed.

Freshly collected root samples were washed with water, gently wiped with absorbent paper to remove excess water, and stored at −40 °C until further analysis. They were individually ground in liquid nitrogen, and 300 mg to 800 mg of frozen powder was placed in a 4 mL glass vial that was then tightly closed with a septum-containing lid. The vial was left to equilibrate for 20 min in a water bath set at 80 °C. VOCs were sampled by exposing an HS-SPME fiber (Supelco, Bellefonte, PA, USA) coated with a 100 μm film of polydimethylsiloxane (PDMS) to the vial headspace for 30 min. The fiber type (100 µM PDMS) was selected based on the recommendations of the VOC analysis supplier. The HS-SPME fiber was introduced hermetically into the vial through the septum. Before each extraction, the fiber was cleared from any volatile contaminant by incubating it for 15 min at 250 °C and by checking the absence of contaminants via GC/MS analysis. Duplicate VOC samplings were consecutively made on each vial and were later analyzed by GC/MS.

The VOCs were desorbed from the HS-SPME fibers by introducing individual fibers directly in the GC/MS injection inlet set at 250 °C for 2 min. GC/MS analyses were performed on a Shimadzu QP2010SE instrument (Marne-la-Vallée, France) fitted with a fused-silica capillary column (30 m × 0.25 mm) coated with a 0.25 μm film of 5% phenyl-arylene and 95% dimethylpolysiloxane (ZB-5ms, Phenomenex, Le Pecq, France) and a quadrupole analyzer. A split injection with a ratio of 5 was used for all samples. The column oven temperature was initially maintained at 70 °C for 3 min, linearly increased to 260 °C at 3 °C min^−1^, and maintained at 260 °C for 10 min. The injection port and ion source temperatures were set at 250 °C and 200 °C, respectively. Helium was used as the carrier gas at a flow rate of 1 mL min^−1^. Electronic impact ionization was performed at 70 eV, and the quadrupole functioned under scan mode, with a scan range of 35–350 *m*/*z* and an acquisition time of 0.20 s. Compounds were identified by their mass spectral and retention index similarities with reference compounds in standard reference databases [38,54].

### 3.4. Hydrodistillation

The hydrodistillation of air-dried HPA-grown roots (116 g) was undertaken with a Clevenger apparatus for 3 h. The resulting distillate was solvent extracted with a mixture of pentane/ether (50/50), and the mass of the EO was measured after solvent evaporation. The EO contents were expressed as % DW (*m*/*m*). The same extraction procedure was applied to air-dried roots of vetiver recovered from fields in Kenya (Green Cycle Consulting, Nairobi, Kenya), Senegal (nursery Naac Baal, Sebikotane, Senegal) and France (Domaine Vidot, Société Horticole du Bassin Plat, The Reunion Island, France) during the same year. For field-grown plants, the engaged dry root masses were 158 g, 171 g and 184 g, respectively.

## 4. Conclusions

In conclusion, this study compared, for the first time, the accumulation of vetiver VOCs in the roots of SG and HPA-grown plants of interest to the perfume industry. A GC/MS analysis of root VOCs sampled by HS-SPME revealed that the soilless HPA plants did not accumulate most of the abundant sesquiterpenes that are responsible for the vetiver fragrance. In agreement with this result, their EO content was much lower. HPA-grown plants also developed longer, thinner, and more tapered roots and a lower root system biomass. This corresponded to an underdevelopment of the pith, central cylinder, and cortex parenchyma. EO-accumulating cells were, therefore, less numerous. They also contained fewer EO vesicles of a smaller diameter. In addition, differences in root-associated microbiota did not explain these differences in root development and VOC accumulation since axenically grown tissue-cultured plants had a phenotype that was more similar to HPA-grown plants. This study paves the way for further studies aiming to improve specialized metabolite production from vetiver via HPA cultivation.

## Figures and Tables

**Table 1:** Vetiver VOCs observed during GC/MS analyses.

N° ^a^	Compound Names ^b^	RI_ZB-5MS_^c^	RI_ref_^d^	In Vitro	HPA	Potting Soil
1	**117** 188 ^e^	1348	-	-	-	×
2	Cyclosativene	1366	1369 ^f^	-	-	×
3	2-epi-α-Funebrene	1379	1380 ^f^	-	×	×
4	β-Elemene	1386	1389 ^f^	-	×	-
5	91 **131** 188 ^e^	1404	-	-	×	-
6	2-epi-β-Funebrene	1411	1411 ^f^	-	×	×
7	β-Cedrene	1419	1419 ^f^	-	×	×
8	γ-Elemene *	1427	1434 ^f^	-	×	-
9	EpiBicyclosesqui-phellandrene	1428	1430 ^g^	-	-	×
10	6,9-Guaiadiene	1440	1442 ^f^	-	-	×
11	Prezizaene *	1446	1444 ^f^	-	×	×
12	Khusimene	1451	1453 ^f^	-	×	×
13	α-Acoradiene	1462	1464 ^f^	-	×	-
14	**119** 202 ^e^	1463	-	-	-	×
15	Dehydro-aromadendrene	1467	1465 ^h^	-	-	×
16	α-Amorphene	1477	1483 ^f^	-	-	×
17	δ-Selinene	1485	1492 ^f^	-	-	×
18	γ-Amorphene	1489	1495 ^f^	-	-	×
19	Isodaucene	1496	1500 ^f^	-	×	-
20	β-Himachalene	1501	1500 ^f^	-	-	×
21	δ-Amorphene *	1518	1511 ^f^	-	-	×
22	41 69 **105** 204 ^e^	1520	-	-	-	×
23	105 133 161 189 **204 **^e^	1529	-	-	-	×
24	105 **161** 204 ^e^	1535	-	-	-	×
25	β-Vetivenene	1550	1554 ^f^	-	-	×
26	Germacrene B	1553	1559 ^f^	-	×	×
27	105 133 **189** 204 ^e^	1558	-	-	-	×
28	**152** 222 ^e^	1570	-	-	-	×
29	187 **202 **^e^	1581	-	-	-	×
30	**108** 204 ^e^	1600	-	-	-	×
31	**187** 202 ^e^	1610	-	-	-	×
32	**81** 222 ^e^	1619	-	-	-	×
33	α-Cadinol *	1651	1652 ^f^	-	-	×
34	epi-Zizanone *	1667	1668 ^f^	-	×	×
35	131 **150** 220 ^e^	1674	-	-	-	×
36	**41** 177 222 ^e^	1684	-	-	-	×
37	**119** 218 ^e^	1689	-	-	-	×
38	41 67 79 **93** 218 ^e^	1691	-	-	×	-
39	**41** 91 222 ^e^	1694	-	-	-	×
40	Vetiselinenol *	1720	1730 ^f^	-	-	×
41	83 **119** 220 ^e^	1728	-	-	-	×
42	Khusimol	1740	1741 ^f^	-	×	×
43	(*E*)-Isovalencenol *	1785	1793 ^f^	-	-	×
44	93 **105** 120 220 ^e^	1789	-	-	-	×
45	β-Vetivone *	1810	1822 ^f^	-	-	×
46	Vetivenic acid	1816	1811 ^f^	-	-	×
47	41 **91** 105 218 ^e^	1822	-	-	-	×
48	α-Vetivone	1835	1842 ^f^	-	-	×
49	91 147 218 ^e^	1893	-	-	-	×

^a^ GC peak number in Figure 2. ^b^ Compound names identified by similarity of the mass spectrum with those from NIST11, NIST11s, or the Adams library and by the similarity of the retention indices with those from the literature. ^c^ Retention indices calculated from a series of C_8_–C_20_ n-alkanes on a ZB-5MS column. ^d^ Retention indices from the literature. ^e^ Unknown compound; most abundant ions of the mass table are displayed with the most intense peak in bold face and with the molecular ion underlined. ^f^ Retention indices from [38]. ^g^ Retention indices from [39]. ^h^ Retention indices from [40]. * Tentatively identified by MS; - compound not detected; × compound detected.

## Data Availability

Not applicable.
